# Maternal profiles and social determinants of malnutrition and the MDGs: What have we learnt?

**DOI:** 10.1186/s12889-016-2853-z

**Published:** 2016-03-02

**Authors:** Edem M. A. Tette, Eric K. Sifah, Edmund T. Nartey, Peter Nuro-Ameyaw, Pricilla Tete-Donkor, Richard B. Biritwum

**Affiliations:** Department of Community Health, School of Public Health, University of Ghana, P.O. Box LG 25, Legon, Accra, Ghana; Princess Marie Louis Children’s Hospital (PML), P.O. Box GP 122, Accra, Ghana; Centre for Tropical Clinical Pharmacology & Therapeutics, School of Medicine and Dentistry, University of Ghana, P. O. Box GP 4236, Accra, Ghana; Department of Nutrition, University of Health and Allied Sciences, Ho, Ghana

**Keywords:** Malnutrition, Social care, HIV, Maternal characteristics, Child, MDGs

## Abstract

**Background:**

Maternal socio-demographic and health profiles are important determinants of malnutrition in children. In the 1990s, malnutrition was associated with low-birth-weight, young mothers and low maternal socio-economic status at Princess Marie Louise Children’s Hospital (PML). It is not known how this has changed by efforts to achieve the Millennium Development Goals. We examined socio-demographic and health profiles of mothers of children with acute malnutrition and those without the condition to identify risk factors for malnutrition and focus on preventive efforts.

**Methods:**

An unmatched case–control study was conducted in 2013 at PML, the largest facility for treating malnourished children in Ghana in 2013. Mothers of children with moderate and severe acute malnutrition were compared with mothers of well-nourished children. Weight-for-height was used to classify malnutrition. Record forms and a semi-structured questionnaire were used for data collection. An analysis was done with Stata 11.0 software.

**Results:**

Altogether, 371 mothers were studied consisting of 182 mothers of malnourished children and 189 mothers of well-nourished children. Mothers of malnourished children were more likely to be unmarried or cohabiting, have lower family incomes, HIV infection and chronic disease. They were less likely to stay with or provide alternative care for their child. Awareness and use of social services, health insurance and a cash transfer programme were low. A remarkable reduction in the number of malnourished children occurred when families earned more than $250 USD a month. Over-nutrition was present in both groups of mothers.

**Conclusion:**

Low family income, unmarried status and type of child care were the main social determinants of malnutrition. There appears to be a reduction in the number of other poor socio-demographic characteristics in both the study and control groups compared to results from a previous study at the same centre, probably because of efforts toward attaining the MDGs. These findings suggest that prevention and optimum management need to involve multidisciplinary teams consisting of health professionals, social workers and/or key workers to enable families at risk to access social care and social protection interventions (MDG 1). This will make the management of malnutrition more effective, prevent relapse, protect the next child and address maternal over-nutrition.

**Electronic supplementary material:**

The online version of this article (doi:10.1186/s12889-016-2853-z) contains supplementary material, which is available to authorized users.

## Background

Malnutrition in childhood is a major risk factor for death and disability worldwide [1, 2]. Children who survive malnutrition are at risk of a cycle of recurrent infection and malnutrition as well as learning disability which reduce their ability to develop to their full potentials and contribute positively to economic development [2, 3]. Although there has been a gradual reduction in the incidence of the condition over the past decade, globally, it is estimated that about 52 million children (8 %) under the age of five years are malnourished and wasted [4]. One of the reasons why the condition has been difficult to control, even though it is largely preventable, is that it is often the result of a constellation of factors which include socio-demographic, economic, health and biologic determinants of both the mother and child [5–10].

Studies have associated malnutrition with maternal illiteracy, lack of education, lack of support in the home, mothers going back to work early and neglect [5–10]. Poverty and low maternal education have also been identified as major determinants of malnutrition in Ghanaian children [9, 10]. These studies also show that social factors are prominent and the predominant factors at play vary from place to place even within the same region. This makes it untenable to prescribe the same intervention for all situations. Therefore, it is important to review the determinants of malnutrition in a locality periodically as countries develop socio-economically.

Over the past decade, there have been several initiatives to reduce child poverty and improve access to education and reproductive health services in order to achieve the Millennium Development Goals (MDG 1, 2, 3, 4 and 5) [11–14]. These goals impact on the nutritional status of children by eradicating hunger and extreme poverty (MDG1), increasing access to basic education (MDG2), empowering women (MDG3) and reducing the number of children orphaned by maternal deaths (MDG5). It is not clear how all these efforts have affected the epidemiology of child malnutrition and in particular the profile of mothers whose children have either moderate acute malnutrition (MAM) or severe acute malnutrition (SAM). We examined maternal socio-economic/demographic and health characteristics of children attending the Princess Marie Louise Children’s hospital to determine the risk factors for malnutrition that are amenable to preventive interventions.

## Methods

### Study setting

Princess Marie Louise (PML) Hospital is a 74 bed children’s hospital situated in the commercial centre of the capital of Ghana, Accra. It hosts the largest centre for treating children with severe malnutrition in Ghana. It attends to approximately 70,000 children at the outpatients department each year. The hospital offers both primary care and specialized paediatric services; thus, the hospital attends to sick children brought in by their parents and referrals from health facilities in other parts of Accra, particularly, children with malnutrition. Occasionally, the referrals come from other regions of the country. In 1933, while working there as an expatriate doctor, Dr Cicely Williams described Kwashiorkor as a form of malnutrition. In 2012, there were 157 inpatient admissions for MAM or SAM at PML with a case fatality rate of 14 %. Malnutrition case management at PML hospital is based on the WHO guidelines.

### Study design and sampling

An unmatched case–control study was conducted at the Princess Marie Louise children’s hospital (PML) between January 2013 and September 2013. This was part of a larger study to determine risk factors for child malnutrition in both mothers and children. Consecutive patients with MAM or SAM were recruited from the outpatients department, nutritional rehabilitation unit, malnutrition ward and other wards, if they met the inclusion criteria and gave consent. These were the cases. Their maternal and social profiles were compared with a group of mothers whose children did not have either MAM or SAM and were labelled as well-nourished. These were the controls. The study also examined child characteristics and feeding practices and related it to the risk of malnutrition. The results are yet to be published.

With an estimated prevalence of 0.09 and a confidence interval of 95 and 5 % percentage points, we estimated a minimum sample size of 126 children and their mothers and targeted 150 children in each group. Due to difficulty in obtaining sufficient patients in the comparison group, recruitment of the comparison group needed to be extended to September 2013. Full details of the study design can be found in the STROBE statement (Additional file 1).

### Study population

Children under the age of 5 years with MAM or SAM, determined by weight for height measurements, their mothers, as well as their unmatched controls and their mothers were studied. Children with a recognised cause of chronic malnutrition such as congenital heart disease, renal failure, sickle cell disease or liver disease and their mothers were excluded from the study groups. Children in the nutritional rehabilitation programme for more than 7 days and their mothers were also excluded. The reason for this was to reduce the likelihood of mothers reporting the advice they had received from the hospital for the study of feeding practices rather than their own feeding practices prior to entering the study. Children who were severely ill were also excluded until they were stable, i.e., if this was within the 7 days.

### Definitions for malnutrition

- Children with an admission weight for height <70 % of the National Centre for Health Statistics (NCHS) median value or below −3 z score with or without bilateral pitting oedema have Severe Acute Malnutrition (SAM).
- Children with a weight for height <80 % but ≥70 % of the National Health Statistics (NCHS) median value or between −2 and −3 z score have Moderate Acute Malnutrition(MAM).
- Children aged 6 months and above with Mid Upper Arm Circumference (MUAC) less than 11.5 cm have Severe Acute Malnutrition (SAM) while those with a Mid Upper Arm Circumference ≥11.5 cm and <12.5 cm have Moderate Acute malnutrition (MAM) [15, 16].

### Measurements

The mid upper arm circumference (MUAC) was initially used as a measurement for identifying and admitting patients with SAM and MAM as is the practice in Ghanaian health facilities. However, only children with a weight for height less than −2 z scores and their mothers were included in the study as malnourished cases. This meant that children who met MUAC criteria but did not meet weight for height criteria and their mothers were excluded from the study and vice versa. Patients with a weight for height greater than −2 z scores presenting to the hospital with other conditions were included in the control group.

Weight measurements of the mothers were done using a UNICEF EKS floor scale. Height measurements were done using a Leicester height measure and recorded to the nearest millimetre. Weight and length/height measurements of the children were done using a Class III infant scale (Seca 334) and a Seca 417 length board or a Leicester height measure. The World Health Organization Anthro-calculator was used to determine the weight for length/height. The research personnel involved in making these measurements were trained in standardised techniques for performing the measurements using a Royal College of Paediatrics and Child Health training video clip.

Body Mass Index which is weight divided by height squared in kg/m^2^ was obtained from the weight and height measurements and further categorized using WHO classification into ≥30 kg/m^2^ Obese, 25.0–29.9 kg/m^2^ Pre-obesity (Overweight), 18.5–24.9 kg/m^2^ Normal and below 18.5 kg/m^2^ Underweight [17].

### Data collection

Data were collected with a semi-structured questionnaire and a data record form. Information on socio-demographic and maternal characteristics was obtained by interviewing the child’s mother or caregiver. Information on maternal age, religion, marital status, employment status, occupation, educational level, family income, number of children and residence were collected. Family income was reported as monthly income because most salaried workers in Ghana are paid at the end of the month. Data were also collected on smoking and drinking habits, problems during pregnancy, maternal illness, child care, support and contact with social services. In addition, information on registration with the National Health Insurance Scheme (NHIS), awareness and use of a cash transfer programme, the Livelihood Empowerment to Alleviate Poverty (LEAP) Programme and the hospitals needy children’s fund was collected.

### Statistical methods

The data was entered into a Microsoft Access (Microsoft Corporation, Redmond, Washington) and analysed using Stata 11.0® (College Station, Texas 77845 USA). Frequencies and means were computed and the data was summarized in tables and graphs. Comparisons and statistical inference were made using the Chi square test and odds ratios to determine the degree of statistical significance of the criteria. Statistical significance was accepted at a 5 % probability level, that is, a p-value of less than 0.05. Bivariate and multivariate logistic regression analyses were also done.

### Ethics

Ethical clearance was obtained from the University of Ghana Medical School’s Ethical and Protocol Review Committee [Protocol Identification Number: MS-Et/M.8-P.5.8/2011-2012] and Ghana Health Service Ethical Review Committee [Protocol Identification Number GHS-ERC 05/07/2012]. Written consent was obtained from the parents (mostly mothers) or carers of the children using consent forms which were signed or thumb printed.

## Results

A total of 371 mothers (or carers) and child pairs were studied, consisting of 182 malnourished children and 189 well-nourished children. The ages of the children ranged between 6 and 51 months among the malnourished cases and 6 and 54 months among the well-nourished controls.

### Socioeconomic and demographic characteristics

**Fig. 1:**
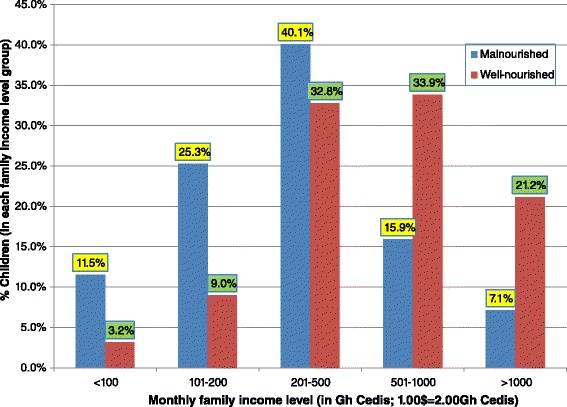
Family income and nutritional status of 371 children attending PML Hospital in Accra, Ghana

Table 1 shows maternal socio-economic and demographic characteristics and its association with malnutrition in the children. The majority of the mothers were between the ages of 20 and 34 years. Mother’s current age, mother’s age at pregnancy, mother’s educational status and mother’s literacy status were associated with malnutrition in the children in the bivariate analysis but were not in the multivariate analysis (Table 1). In the multivariate analysis, the odds of a child being malnourished was higher with mothers who were single or cohabiting compared with married mothers (adjusted OR 2.43 [95 % CI, 1.05–5.61], *p* = 0.038 and adjusted OR 2.24 [95 % CI, 1.16–4.34], *p* = 0.016 respectively). Similarly, the odds of a child being malnourished was higher in mothers with a family income of ≤500 Ghana Cedis ($250 USD) compared with mothers with a family income of >500 Ghana cedis (adjusted OR, 2.73 [95 % CI, 1.61–4.65], *p* < 0.001) (Table 1). The majority of single mothers, 92.5 %, reported a family income of 500 Ghana Cedis or less, whereas only 50 % of mothers who were married reported a family income of 500 Ghana Cedis or less (*p* < 0.001). Similarly, the majority of women who were cohabiting (69 %) reported a family income of 500 Ghana Cedis or less. The difference between the latter and married mothers was statistically significant (*p* < 0.001). Figure 1 shows the relationship between family income and nutritional status. A total of 7.1 % of the malnourished children had a family income above 1000 Ghana cedis compared with 21.2 % in the well-nourished group.

**Table 1:** Maternal socio-economic and demographic characteristics associated with malnutrition in 371 children attending PML hospital in Accra, Ghana

Characteristic	Nutritional status of child					
	Malnourished n, %	Well-nourished n, %	Crude OR [95 % CI]	p-value	Adjusted OR [95 % CI]^*^	p-value
Mother’s current age (*N* = 357)						
≤19 years	8 (4.7)	2 (1.1)	4.39 [0.85–42.97]	0.045	1.00 [0.14–7.22]	0.999
≥35 years	30 (17.5)	38 (20.4)	0.87 [0.49–1.53]	0.599	1.07 [0.57–1.98]	0.841
20–34 years	133 (77.8)	146 (78.5)	1.00		1.00	
Mother's age during pregnancy (*N* = 357)						
≤19 years	19 (11.1)	8 (4.3)	2.78 [1.12–7.54]	0.015	1.50 [0.47–4.73]	0.492
≥20 years	152(88.9)	178 (95.7)	1.00		1.00	
Mother’s marital status (*N* = 346)						
Single	29 (17.6)	11 (6.1)	4.21 [1.91–9.79]	<0.001	2.43 [1.05–5.61]	**0.038**
Separated/Divorced/widowed	9 (5.5)	9 (5.0)	1.60 [0.61–4.18]	0.340	0.54 [0.16–1.82]	0.324
Co-habiting	40 (24.2)	22 (12.2)	2.90 [1.56–5.48]	<0.001	2.24 [1.16–4.34]	**0.016**
Married	87(52.7)	139 (76.8)	1.00		1.00	
Mother's educational status (*N* = 365)						
None	25 (14.0)	12 (6.5)	7.18 [2.35–22.50]	<0.001	2.73 [0.75–9.96]	0.128
Basic	112 (62.6)	93 (50.0)	4.19 [1.80–10.37]	<0.001	2.10 [0.81–5.45]	0.126
Secondary/Vocational	33 (18.4)	50 (26.9)	2.27 [0.90–6.11]	0.059	1.35 [0.50–3.62]	0.549
Tertiary	9 (5.0)	31 (16.7)	1.00		1.00	
Mother can read and write (*N* = 368)						
No	81 (44.8)	49 (26.2)	2.28 [1.44–3.63]	<0.001	1.09 [0.58–2.03]	0.794
Yes	100 (55.2)	138 (73.8)	1.00		1.00	
Monthly family income (*N* = 371)						
≤ 500 Gh Cedis^a^	140 (76.9)	85 (45.0)	4.08 [2.55–6.56]	<0.001	2.73 [1.61–4.65]	**<0.001**
> 500 Gh Cedis^a^	42 (23.1)	104 (55.0)	1.00		1.00	
Receives extra financial support due to inadequate family income (*N* = 371)						
Yes	44 (24.2)	24 (12.7)	2.19 [1.23–3.96]	0.004	1.47 [0.77–2.82]	0.241
No	138 (75.8)	165 (87.3)	1.00		1.00	

^a^1.00$ = 2.00GH Cedis; ^*^Varibales with *p* < 0.2 in the bivariate analysis were entered into the multivariate analysis model; *OR* Odds ratio, *CI* Confidence interval

### Maternal health and malnutrition

Table 2 shows the association between maternal health and malnutrition. HIV infection was reported in 13 (7.1 %) of the cases and 3 (1.6 %) of the controls. Five of these mothers were diagnosed during pregnancy. In the multivariate analysis, maternal HIV-positive status and the presence of a chronic illness in a mother were associated with higher odds of malnutrition in a child (adjusted OR 5.41 [95 % CI, 1.29–22.77], *p* = 0.021; adjusted OR 6.51 [95 % CI, 1.27–33.47], *p* = 0.025 respectively). However, a mother’s BMI or complication during pregnancy was not associated with malnutrition in both the bivariate and multivariate analyses (Table 2).

**Table 2:** Maternal health characteristics associated with malnutrition in 371 children attending PML hospital in Accra, Ghana

Characteristic	Nutritional status of child					
	Malnourished n, %	Well-nourished n, %	Crude OR [95 % CI]	p-value	Adjusted OR [95 % CI]^*^	p-value
Mother’s marital status (*N* = 346)						
Single	29 (17.6)	11 (6.1)	4.21 [1.91–9.79]	<0.001	2.70 [1.12–6.51]	0.027
Separated/Divorced/widowed	9 (5.5	9 (5.0)	1.60 [0.61–4.18]	0.340	0.14 [0.02–0.99]	0.048
Co-habiting	40 (24.2)	22 (12.2)	2.90 [1.56–5.48]	<0.001	2.23 [1.13–4.40]	0.021
Married	87(52.7)	139 (76.8)	1.00		1.00	
Monthly family income (*N* = 371)						
≤500 Gh Cedis^a^	140 (76.9)	85 (45.0)	4.08 [2.55–6.56]	<0.001	3.05 [1.75–5.31]	<0.001
>500 Gh Cedis^a^	42 (23.1)	104 (55.0)	1.00		1.00	
Mother's BMI category (*N* = 336)						
Normal Weight	89 (55.3)	66 (37.9)	1.01 [0.14–6.20]	0.989	-	-
Over-weight/Obese	68 (42.2)	105 (60.3)	0.49 [0.07–2.98]	0.345	-	-
Under-weight	4 (2.5)	3 (1.7)	1.00			
Mother’s BMI category (*N* = 347)						
Yes	22 (13.5)	24 (13.0)	1.04 [0.53–2.03]	0.901	-	-
No	141 (86.5)	160 (87.0)	1.00			
Maternal HIV status (*N* = 288)						
Positive	13 (7.1)	3 (1.6)	5.92 [1.57–32.91]	0.002	5.41 [1.29–22.77]	0.021
Negative	115 (63.2)	157 (83.1)	1.00		1.00	
Presence of chronic illness in mother (*N* = 371)						
Yes	10 (5.5)	3 (1.6)	3.60 [0.91–20.65]	0.041	6.51 [1.27–33.47]	0.025
No	172 (94.5)	186 (98.4)	1.00		1.00	

^a^1.00$ = 2.00GH Cedis; ^*^Varibales with *p* < 0.2 in the bivariate analysis were entered into the multivariate analysis model in addition to marital status and monthly family income; *BMI* Body mass index, *OR* Odds ratio, *CI* Confidence interval

### Child care and malnutrition

Table 3 shows the association between types of child care and malnutrition in children. The majority of mothers stayed with or cared directly for their children (91.2 % in cases and 97.4 % in controls). Having problems with child care or directly taking care of a child was not associated with malnutrition in the multivariate analysis, although both variables were associated with malnutrition in the bivariate analysis (Table 3). Similarly, the odds of malnutrition was higher in children whose mothers took them to work or stayed at home with them compared with mothers who left their children at a crèche or nursery (adjusted OR, 2.90 [95 % CI, 1.27–6.62], *p* = 0.012; adjusted OR, 4.46 [95 % CI, 1.90–10.48], *p* = 0.001 respectively) (Table 3).

**Table 3:** Maternal child caring characteristics associated with malnutrition in 371 children attending PML hospital in Accra, Ghana

Characteristic	Nutritional status of child					
	Malnourished n, %	Well-nourished n, %	Crude OR [95 % CI]	p-value	Adjusted OR [95 % CI]^*^	p-value
Mother’s marital status (*N* = 346)						
Single	29 (17.6)	11 (6.1)	4.21 [1.91–9.79]	<0.001	2.45 [1.09–5.53]	0.031
Separated/Divorced/widowed	9 (5.5	9 (5.0)	1.60 [0.61–4.18]	0.340	0.99 [0.31–3.18]	0.987
Co-habiting	40 (24.2)	22 (12.2)	2.90 [1.56–5.48]	<0.001	2.24 [1.18–4.26]	0.014
Married	87(52.7)	139 (76.8)	1.00		1.00	
Monthly family income (*N* = 371)						
≤500 Gh Cedis^a^	140 (76.9)	85 (45.0)	4.08 [2.55–6.56]	<0.001	2.81 [1.65–4.78]	<0.001
>500 Gh Cedis^a^	42 (23.1)	104 (55.0)	1.00		1.00	
Mother directly cares for/stay with child (*N* = 371)						
No	16 (8.8)	5 (2.6)	3.55 [1.20–12.62]	0.010	2.08 [0.60–7.18]	0.245
Yes	166 (91.2)	184 (97.4)	1.00		1.00	
Having problems with child care (*N* = 371)						
Yes	94 (51.6))	52 (27.5)	2.81 [1.79–4.44]	<0.001	1.57 [0.92–2.67]	0.100
No	88 (48.4)	137 (72.5)	1.00		1.00	
How mother cares for child during the day (*N* = 368)						
Takes child to work	61 (33.7)	63 (33.7)	3.05 [1.42–6.80]	0.002	2.90 [1.27–6.62]	0.012
Leaves child with care giver	39 (21.6)	50 (26.7)	2.46 [1.10–5.69]	0.017	2.03 [0.86–4.79]	0.106
Mother is with child at home	68 (37.6)	33 (17.7)	6.50 [2.91–14.93]	<0.001	4.46 [1.90–10.48]	0.001
Leaves child at crèche/nursery	13 (7.2)	41 (21.9)	1.00		1.00	

^a^1.00$ = 2.00GH Cedis; ^*^Varibales with *p* < 0.2 in the bivariate analysis were entered into the multivariate analysis model in addition to marital status and monthly family income; *OR* Odds ratio, *CI* Confidence interval

### Social support systems

Table 4 shows the social support systems in place for mothers to access and their association with malnutrition in children. Only 3.3 % (*n* = 6) of the cases and 1.1 % (*n* = 2) of the controls were aware of the LEAP programme, while five (5) of the malnourished children had benefitted from the hospital’s needy fund. The National Health Insurance Scheme (NHIS) was patronised by 56.6 % (*n* = 103) of the malnourished group and 54 % (*n* = 102) of the well-nourished group. In the bivariate analysis, not having heard of social services had higher odds of malnutrition in children compared with mothers who have heard of such services (OR, 1.61 [95 % CI, 1.04–2.49], *p* = 0.026).

**Table 4:** Social support systems and its association with malnutrition in 371 children attending PML hospital in Accra, Ghana

Characteristic	Nutritional status of child			
	Malnourished n, %	Well-nourished n, %	OR [95 % CI]	p-value
Ever heard of social services (*N* = 371)				
No	118 (64.8)	101 (53.4)	1.61 [1.04–2.49]	0.026
Yes	64 (35.2)	88 (46.6)	Ref	
Seen Social service for help (*N* = 371)				
No	178 (97.8)	187 (98.9)	0.48 [0.43–3.37]	0.384
Yes	4 (2.2)	2 (1.1)	Ref	
Ever heard of LEAP programme (*N* = 371)				
No	176 (96.7)	187 (98.9)	0.31 [0.03–1.79]	0.138
Yes	6 (3.3)	2 (1.1)	Ref	
Ever benefited from hospital needy fund (*N* = 371)				
Yes	5 (2.7)	-	Not estimable	<0.001
No	177 (97.3)	189 (100)	Ref	
Child had registered with NHIS before hospital visit (*N* = 371)				
No	79 (43.4)	87 (46.0)	0.90 [0.58–1.38]	0.611
Yes	103 (56.6)	102 (54.0)	Ref	

*OR* Odds ratio, *CI* Confidence interval, *LEAP* Livelihood empowerment against poverty, *NHIS* national health insurance scheme; 1 malnourished child was reported to be a beneficiary of the LEAP programme

### NHIS enrolment

Table 5 shows reasons for non-enrolment in the NHIS and problems encountered with child care. The most cited reasons for non-enrolment on the NHIS for malnourished children was “it does not work” (49.4 %, *n* = 39), whereas for well-nourished children, there was no satisfactory reason (67.8 %, *n* = 59). Financial problems was the most reported problem with child care in both cases (91.5 %, *n* = 86) and controls (65.4 %, *n* = 34).

**Table 5:** Reasons for non enrolment in NHIS and problems encountered in child caring

Characteristic	Nutritional status of child	
	Malnourished n, %^a^	Well-nourished n, %^a^
Reason for non-enrolment in NHIS		
About to register	-	4 (4.6)
Forgetfulness	7 (8.9)	1 (1.1)
It doesn’t work	39 (49.4)	3 (3.4)
It is expensive	14 (17.7)	7 (8.0)
It is not necessary	2 (2.5)	3 (3.4)
It takes too long to be attended to	1 (1.3)	2 (2.3)
It takes too long to get registered	6 (7.6)	1 (1.1)
No satisfactory reason	28 (35.4)	59 (67.8)
Registered but card not ready	13 (16.5)	5 (5.7)
Wanted to do it but not yet	5 (6.3)	2 (2.3)
Type of problem with child care		
Disability	2 (2.1)	2 (3.8)
Financial	86 (91.5)	34 (65.4)
Housing	6 (6.4)	5 (9.6)
Marital	23 (24.5)	14 (26.9)
Paying hospital bills	17 (18.1)	9 (17.3)
Retrieving child support from partner	31 (33.0)	6 (11.5)
Unemployment	45 (47.9)	20 (38.5)

^a^%s may add up to >100; *NHIS* National health insurance scheme

## Discussion

### Socio-demographic and health characteristics

This study showed that childhood malnutrition in this population was associated with mothers who were single, or cohabiting with a partner. This is, most likely, related to lower family income because, while only half (50 %), of mothers who were married reported a family income of 500 Ghana Cedis or less, the majority (92.5 %) of single mothers reported a family income of 500 Ghana Cedis or less. The difference was statistically significant (*p* < 0.001). The finding is similar to studies done in Nigeria [6, 8] and Malawi [7] but is at variance with other studies from Ethiopia and Nigeria which did not find a statistically significant association between single motherhood and malnutrition [6, 18, 19]. Therefore, interventions specifically targeted at supporting single mothers are likely to prevent malnutrition. This will not only improve maternal well-being but will ultimately protect against child mortality, which is strongly associated with malnutrition in this and other settings (MDG4) [20].

The previous study conducted at PML hospital and reported in 1998 by Rikimaru et al. [5] found that malnutrition was associated with young mothers, low-birth-weight, lower educational levels and certain occupations. In this study, malnutrition was not associated with teenage pregnancy or lower educational levels after multivariate analysis. However, the proportion of teenage pregnancies in this study is considerably low at 11.1 % in the malnourished group and 4.3 % in the well nourished group compared with 35 % in the severely malnourished group and 20 % in the normal group in the previous study which examined teenagers at the time of birth. This could be an effect of MDG 5.

Malnutrition has been associated with low maternal education in several studies done in Ghana [5, 9, 10, 21]. The study by Rikimaru et al. at PML using weight-for-age criteria found that 40 % of the mothers of severely malnourished children had no education, whereas only 22.4 % of well-nourished children had mothers with no education [5]. In our study, which includes both moderate and severely malnourished children, we found that 14.0 % of mothers of malnourished children had no formal education compared to 6.5 % of mothers of well-nourished children. Thus, the general proportion of mothers with no formal education seemed to have dropped remarkably. These findings are, most likely, a positive reflection of efforts to achieve universal primary education outlined in MDG 2 [14]. However, the difference between the proportion of mothers with basic education in the study by Rikimaru et al. and this study was only minimal. That is, 58.3 % among the cases and 53.1 % among the controls were recorded in the study by Rikimaru et al. as against the recorded percentages of 62.6 % among the cases and 50.0 % among the controls in this study.

We also found that basic education was the predominant educational attainment in the malnourished group recorded by 112 mothers (62.6 %). The association between educational level and malnutrition was not significant in the multivariate analysis (Table 1). This is probably because the effect is dependent on the family income as giving one’s child nutritious food is ultimately dependent on how much money is available. If this is so, then it further suggests that receiving universal basic education alone as currently provided by MDG 2 is not sufficient for protection against malnutrition unless it is associated with gaining employable skills which can bring in income. Thus, ultimately, it is the quality of education and the income it provides that matters. Similarly, an analysis of the relationship between employment and malnutrition from this study, presented elsewhere, found a significant relationship between malnutrition and unemployment at the bivariate level but not at the multivariate level [22]. So it appears that the level of income earned from employment is similarly important in preventing malnutrition. Although it has been suggested that improving the quality of nutrition education in school may empower future mothers sufficiently to prevent the condition when they become mothers themselves (MDG 3), the evidence for this is currently limited [23]. Illiteracy was common in mothers of malnourished children, in spite of the majority having received basic education, but was not associated with malnutrition in the multivariate analysis though it has been associated with malnutrition in other studies [6, 18, 19].

Although there was no relationship between BMI and child malnutrition in this study, the level of overweight and obesity in both groups of mothers at 42 % among the cases and 61 % among the controls, is alarming, especially given that most of the mothers of malnourished children are from low income families. This finding is similar to the finding of others who have reported that malnutrition in children is associated with obesity and overweight mothers in countries undergoing nutrition transition [24–26]. We did not establish statistical significance but it calls for a close link between nutrition services for mothers and children so that they can both be managed together.

### Child care

Malnutrition was prevalent in children whose mothers took them to work and those who stayed with their children at home compared with children whose mothers took them to crèche or nursery. This may be related to poverty as mothers who tend to stay at home with their children are often unemployed, while those who take their children to work may be involved in petty trading. Conversely, the protective effect of going to crèche or nursery may be related to better family income since families who can afford crèche or nursery are often financially endowed. On the other hand, it can mean that the children are better fed at these facilities, in which case, it has the potential for reducing the effects of malnutrition due to neglect through the provision of an alternate caregiver. These findings are similar to studies done in Nigeria, which associated child malnutrition with poverty, not living with one’s parents among other factors [6, 8].

### Family financial circumstances

The study showed that mothers of malnourished children had lower incomes and were more likely to receive additional support (Table 2). They reported more problems with child care resulting from unemployment, financial problems, marital problems, difficulty in getting support from partners and paying hospital bills. Malnutrition was associated with family income levels of ≤500 Ghana cedis. The condition has been associated with relative household poverty in both low-income and middle-income countries [25]. Results from this study agree with previous work which showed that low income was associated with childhood malnutrition in Ghana [9, 10]. In addition, the proportion of malnourished children decreased as family income rose above 500 Ghana cedis ($250 USD). This suggests that one should aim at incomes above 500 Ghana cedis ($250 USD) to prevent malnutrition in children (Fig. 1), although it may depend on other factors such as family size, which were not captured here. Nonetheless, it has implications for the cash transfer programme currently in place to alleviate extreme poverty.

### Social support systems

Cash transfer programmes and ways of improving income have been heralded as important interventions to control malnutrition [13, 23, 27, 28]. At the time of the study, in 2013, the Livelihood Empowerment Against Poverty (LEAP) programme offered cash valued at 20 Ghana Cedis ($10 USD) every two months to each beneficiary. This programme which is still running is targeted at extremely poor households as part of the efforts to achieve MDG 1 [13, 28]. The majority of caregivers of both malnourished children and well nourished children were unaware of the cash transfer programme and only one of the mothers with a malnourished child was a beneficiary of the programme.

Anecdotal evidence suggests that some of the families in this study may not qualify for this cash transfer programme since the LEAP programme aims at extremely poor households defined by a proxy measure test. Even if they do qualify, the transferred cash is nowhere near the $250 USD a month shown by this study to reduce the prevalence of malnutrition substantially. The government has recently launched a new version of the programme, LEAP 1000, specifically targeting pregnant women and children. The amount of cash transferred in cedis has also increased. Hopefully, this will impact on malnutrition in children. Nonetheless, we are recommending a separate cash transfer programme for children with malnutrition together with opportunities for their mothers to be empowered through training in income generating activities and employable skills.

The National Health Insurance Scheme established through an Act of parliament offers free medical care and enables medical conditions such as infections and diarrhoea which predispose to malnutrition to be treated early by removing financial barriers to medical care [29]. Under the Act, children under the age of 18 years do not pay any premium if their parents or guardians are registered with the scheme. However, they have to be registered to have access to the facility. There was no significant difference in the way the National Health Insurance Scheme (NHIS) was taken up by both the mothers of the malnourished (56.6 %) and well nourished children (54.0 %) (*p* = 0.611) as the data showed that uptake was average in both groups. Low registration has been reported in other settings [14].

It may be necessary to facilitate access of the insurance to all children by ensuring that newborns are registered before they leave the hospital and by making registration points available at health facilities. Failure to register one’s child after registration has been facilitated in this way could be as a regarded as a criterion for neglect and made to attract a penalty. Further studies are needed to determine the contribution of neglect to the aetiology of malnutrition in this setting and the role of crèches or nurseries since we may be able to use the law to prevent some cases and possibly avert child deaths (MDG 4).

Over the years, it has become increasingly clear that many of the health problems of children including child mortality are related to underlying social problems [30]. Unfortunately, although social services can play a key role in dealing with the problem of childhood malnutrition, only about a third (35.5 %) of the mothers of malnourished children were aware of the service and of these only 4 (2.2 %) had seen social services for help (Table 3). Most of the carers of malnourished children reported social problems that were amenable to services offered by robust social welfare services. These include access to child care and nursery services, parenting classes, counselling, accessing funds for needy children, addressing neglect and providing assistance in obtaining funds for child care from partners through the family tribunals and courts. They can also assist mothers gain access to income generating activities by linking the mothers to non-governmental organizations (NGO’s) and development experts who do this.

Currently, social welfare services are not directly involved in the management of malnourished children, except when it comes to accessing funds to buy medication that is not supplied on the NHIS or to settle unpaid bills. We are proposing that every child with moderate and severe malnutrition needs to have a social worker or a key worker who can link them to these services. There has to be a paradigm shift to make the management of moderate and severe acute malnutrition a multidisciplinary effort involving health professionals such as a doctor, a dietician or nutritionist, a social worker and/or a key worker together with the child and his/her parents. By doing this, both the medical and socio-economic issues enshrined in malnutrition can be tackled concurrently and their effects minimised to prevent relapse and malnutrition in the next child. Though multidisciplinary working is limited in many developing countries, one can develop partnerships and learn from the experience of others such as the Royal College of Paediatrics and Child Health in the UK, who have managed to incorporate this into the care of children with disability and safeguarding (MDG 8) [31, 32].

In order to do this, social services in Ghana and some of the other developing countries will have to be strengthened since it has been reported that these services are often uninformed, ill-equipped, under-developed and not well resourced to do what they are trained to do [33]. Therefore, while great strides have been made over the years in improving the clinical management of malnutrition, more work needs to be done to prevent the condition [34]. As we approach the era of Sustainable Developmental Goals (SDG’s), multidisciplinary working in child health which brings the MDGs together and treats the “whole” child should be our goal.

There were some limitations to this study. Some mothers did not answer all the questions so we had some missing data. The controls were not matched to the cases. We also recognise that although it was expedient to classify children who do not meet the criteria for SAM and MAM as “well-nourished”, they may include some children previously labelled as mild malnutrition using previous criteria.

## Conclusion

Malnutrition was associated with single-motherhood, mothers who were cohabiting, low monthly family income of ≤500 Ghana cedis and mothers with HIV infection or suffering from chronic illness. Taking a child to nursery was protective. Although malnutrition was not associated with low maternal education and teenage pregnancy as previously reported at PML, there appears to be a reduction in the proportions of reported cases of both variables which could be an effect of the MDGs. Social protection interventions such as NHIS and the LEAP programme were not well patronised and the effect of nutrition transition was evident. Thus, in order to prevent and minimise the consequences of childhood malnutrition, efforts must be made to prevent HIV and to enact social policies to tackle poverty. A multidisciplinary approach to management involving health professionals, social workers and key workers will go a long way to prevent relapse, protect the next child and ensure that families are not plagued with a double burden of disease.

## Additional file

Additional file 1:
**STROBE Statement on study.** (DOCX 30 kb)

## Abbreviations

MAM: moderate acute malnutrition
MDGs: Millennium Development Goals
MUAC: mid-upper arm circumference
SAM: severe acute malnutrition
PML: Princess Marie Louise Children’s Hospital
